# Low Grade Endotoxemia and Oxidative Stress in Offspring of Patients with Early Myocardial Infarction

**DOI:** 10.3390/antiox12040958

**Published:** 2023-04-19

**Authors:** Bianca Laura Cinicola, Ilaria Maria Palumbo, Arianna Pannunzio, Roberto Carnevale, Simona Bartimoccia, Vittoria Cammisotto, Martina Capponi, Giulia Brindisi, Francesca Salvatori, Francesco Barillà, Francesco Martino, Alessandra D’Amico, Roberto Poscia, Alberto Spalice, Anna Maria Zicari, Francesco Violi, Lorenzo Loffredo

**Affiliations:** 1 Department of Maternal Infantile and Urological Sciences, Division of Pediatric Allergology and Immunology, Sapienza University of Rome, Viale Regina Elena 324, 00161 Rome, Italy; 2 Department of Molecular Medicine, Sapienza University of Rome, Viale Regina Elena 391, 00161 Rome, Italy; 3 Department of Clinical, Internistic, Anaesthetic and Cardiovascular Sciences, Sapienza University of Rome, Viale del Policlinico 155, 00161 Rome, Italy; 4 Department of Medical-Surgical Sciences and Biotechnologies, Sapienza University of Rome, Corso della Repubblica, 79, 04100 Latina, Italy; 5 IRCCS Neuromed, Località Camerele, 86077 Pozzilli, Italy; 6 Unit of Cardiology, University Hospital “Tor Vergata”, 00133 Rome, Italy; 7 Department of Pediatrics and Pediatric Neuropsychiatry, Sapienza University of Rome, Piazzale Aldo Moro, 5, 00185 Rome, Italy; 8 Department of Movement, Human and Health Sciences, University of Rome “Foro Italico”, Piazza Lauro De Bosis, 15, 00135 Rome, Italy; 9 Unita di Ricerca Clinica e Clinical Competence-Direzione Generale, AOU Policlinico Umberto I, 00161 Rome, Italy; 10 Mediterranea Cardiocentro-Napoli, 80122 Naples, Italy

**Keywords:** myocardial infarction, platelet activation, LPS, gut microbiota, NADPH oxidase, NOX-2, oxidative stress, thromboxane

## Abstract

*Background and aims*: Offspring of patients with early myocardial infarction are at higher cardiovascular risk, but the underlying physio-pathological mechanism is unclear. NADPH oxidase-type 2 (NOX-2) plays a pivotal role as mediator of oxidative stress and could be involved in activating platelets in these patients. Furthermore, altered intestinal permeability and serum lipopolysaccharide (LPS) could be a trigger to promote NOX-2 activation and platelet aggregation. This study aims to evaluate the behavior of low grade endotoxemia, oxidative stress and platelet activation in offspring of patients with early myocardial infarction. *Methods:* We enrolled, in a cross-sectional study, 46 offspring of patients with early myocardial infarction and 86 healthy subjects (HS). LPS levels and gut permeability (assessed by zonulin), oxidative stress (assessed by serum NOX-2-derived peptide (sNOX2-dp) release, hydrogen peroxide (H_2_O_2_) production and isoprostanes), serum nitric oxide (NO) bioavailability and platelet activation (by serum thromboxane B2 (TXB2) and soluble P-Selectin (sP-Selectin)) were analyzed. *Results:* Compared to HS, offspring of patients with early myocardial infarction had higher values of LPS, zonulin, serum isoprostanes, sNOX2-dp H_2_O_2_, TXB2, p-selectin and lower NO bioavailability. Logistic regression analysis showed that the variables associated with offspring of patients with early myocardial infarction were LPS, TXB2 and isoprostanes. The multiple linear regression analysis confirmed that serum NOX-2, isoprostanes, p-selectin and H_2_O_2_ levels were significantly associated to LPS. Furthermore, serum LPS, isoprostanes and TXB2 levels were significantly associated with sNOX-2-dp. *Conclusions:* Offspring of patients with early myocardial infarction have a low grade endotoxemia that could generate oxidative stress and platelet activation increasing their cardiovascular risk. Future studies are needed to understand the role of dysbiosis in this population.

## 1. Introduction

Cardiovascular disease is one of the leading causes of death in the world, with 7 million people being diagnosed with acute coronary syndrome (ACS) every year [1]. Some risk factors are well established, like hypertension, cigarette smoking, hypercholesterolemia, obesity and diabetes mellitus, but others are still debated [2]. Among the emerging risk factors, the Framingham study showed an increased cardiovascular risk in people with a family history of coronary artery disease (CAD) [2], even in the absence of traditional risk factors. In addition, a previous meta-analysis showed that this risk increased significantly when the parents’ myocardial infarction occurred under the age of 50 years [3]. The physio-pathological causes of this increased cardiovascular risk have been investigated in recent years and oxidative stress is considered one of the main factors implicated in this process [3]. Oxidative stress is the result of the imbalance between the production of reactive oxygen species (ROS) and antioxidants [3,4]. It is part of the response mediated by innate immunity, which is the first line of defense against pathogens and is involved in the mechanisms responsible for atherosclerosis and thrombosis [3,4]. The reactive species produced by several cell types, like macrophages and neutrophils, cause damage to microbial structures, but if this process is dysregulated it can damage the host cells too [3,4]. Indeed, the activation of the NADPH oxidase-type 2 (NOX-2), an enzyme that produces ROS and interacts with arachidonic acid, contributes to platelet activation [3,4].

The NOX2 enzyme is composed of several subunits that interact and forms an active enzyme complex. In particular, the two NOX2 subunits, gp91phox and p22phox are transmembrane proteins and together compose the flavocytochrome (cit) b558, while p47phox, p67phox, and p40phox and small G-proteins, Rac1 (in monocytes) or Rac2 (in neutrophils) are cytosolic protein subunits. Under unstimulated conditions, the regulatory subunits, p40phox, p47phox and p67phox, are localized in the cytosol. Upon stimulation, p47phox undergoes phosphorylation, and the entire complex translocates to the membrane and associates with cytb558 to form the active oxidase able to generate ROS.

The principal pathway implicated in p47phox phosphorylation involves protein kinase C (PKC) isoforms (δ, β, α, ζ) that, when activated, phosphorylates specific serine residues of p47phox such as Ser304, Ser315, Ser320 and Ser328 [5].

Arachidonic acid acts synergistically with PKC and increases the activation of NOX2, potentiating the p47phox binding to p22phox [6]. 

Several studies have shown that the activation of the PKC/Nox signaling complex regulates ROS production in several type of cells including phagocytes, cardiomyocytes, and platelets, and is involved in more pathophysiological conditions such as cardiovascular diseases [7].

Furthermore, the alteration of intestinal permeability increases inflammation through the production of metabolites, like trimethylamine N-oxide (T-MAO), which have a pro-thrombotic action [8,9,10,11].

LPS is an endotoxin derived from the membrane of Gram-negative bacteria. Impaired intestinal permeability may result in low grade endotoxemia [12]. This is a condition characterized by high serum lipopolysaccharides (LPS), approximately 50 pg/mL, which is about two orders of magnitude lower than that found in sepsis condition [12]. The translocation of LPS in the systemic circulation leads to multiple consequences that can affect conditions predisposing a chronic inflammatory state that can increase cardiovascular risk [12].

LPS plays a major role as mediator of inflammatory processes; LPS is carried in the systemic circulation by LPS binding protein (LBP) or lipoproteins and interacts with some innate receptors of the immune system, such as toll-like receptors (TLRs), expressed by several cells such as dendritic cells, macrophages, T and B lymphocytes and epithelial and endothelial cells and fibroblasts [13].

By binding to TLR4, LPS prompts the intracellular transcription of inflammatory pathways and NADPH oxidase activation [14,15], resulting in platelet activation, thrombosis and atherosclerosis [12,16].

The artery damage mediated by LPS occurs through its binding to the TLR4 located in leukocytes, endothelial cells and platelets [12]. The cascade triggered by this process involves the phosphorylation of Toll/Interleukin-1 receptor (TIR) domain-containing adapter protein (TIRAP) and the recruitment of the primary response protein of differentiation Myeloid 88 (MyD88) in the cytoplasmic domain of TLR4. Downstream signaling induces activation of nuclear transcription factor κB (NF-κB), which increases the production of pro-inflammatory cytokines, such as IL-8 and tumor necrosis factor (TNF), causing an inflammatory response that can lead to an increase in the atherosclerotic plaque but also can lead to serious consequences such as its erosion and rupture [12]. Moreover, the activation of TLR4 contribute to the risk of thrombosis, since in endothelial cells, it induces the release of von Willebrand factor (vwf) and factor VIII (FVIII) through formation and secretion of Weibel–Palade bodies (wpb) and upregulation of tissue factor secretion (TF), which converts the X factor (FX) into the activated X factor (fxa) to generate thrombin [12]. In addition, the activation of leukocytes, mediated by LPS, with the formation of extracellular neutrophil extracellular traps (NETS), causes activation of coagulation cascade factors and thrombosis [12].

LPS induces increased oxidative stress by NADPH oxidase 2 (NOX2) activation; as consequence of NOX2-generated oxidative stress, an increased oxidation of LDL leads to the formation and progression of atherosclerosis [12].

It was recently shown that offspring of patients with early myocardial infarction have higher oxidative stress and platelet activation than offspring of healthy parents [3]. However, studies on the role of altered intestinal permeability and LPS in this population are lacking. Therefore, this study aimed to assess whether offspring of patients with early myocardial infarction have low grade endotoxemia associated with increased oxidative stress and platelet activation.

## 2. Materials and Methods

One hundred thirty-two consecutive subjects, including 46 offspring of patients with early myocardial infarction and 86 healthy subjects (HS) matched for age and gender, were enrolled. Healthy subjects (HS) were recruited through a screening program for metabolic diseases in childhood at the Pediatric Unit of Sapienza University of Rome. They didn’t have a history of any specific metabolic disease. Offspring of patients with early myocardial infarction were identified from patients discharged by the Cardiology Unit of Sapienza University with acute myocardial infarction diagnosis under the age of 50 years. Body mass index (BMI) was determined according to sex- and age-specific growth charts; obesity was defined as a BMI ≥ 95th percentile [17]. Hypercholesterolemia was classified as the presence of total cholesterol > 90th age- and sex-specific percentile and without clear family transmission [18]. Hypertension was defined as systolic and/or diastolic blood pressure > 95th percentile [19]. Hyperglycemia was defined as a fasting blood glucose level ≥ 100 mg/dL [20]. If the subject had smoked even one cigarette in the last three months, the subject was considered as an active smoker. The exclusion criteria were: (1) the presence of hypothyroidism, renal disease, malignancy, treatment with immuno-suppressive drugs, connective tissue diseases and acute illness; (2) familial hypercholesterolemia; and (3) type 1 diabetes. None of the subjects had clinical evidence of cardiovascular disease (as shown by clinical history, physical examination or electrocardiogram). Patients with hypercholesterolemia had not taken any lipid-lowering agent. Informed written consent was obtained by each subject. The study was conformed to the ethical guidelines of the Declaration of Helsinki and was approved by the local Ethical Committee.

Between 8:00 a.m. and 9:00 a.m., the subjects underwent routine biochemical evaluations, including fasting total cholesterol and glucose. After overnight fasting (12 h) and supine rest for at least 10 min, blood samples were collected in vacutainers (Vacutainer Systems, Belliver Industrial Estate, Plymouth, United Kingdom) and centrifuged at 5000× *g* for 10 min to obtain supernatant, which was stored at 80 °C until use. Serum total cholesterol was measured by an AU 560 autoanalyzer (Olympus Optical Co., Sizuoka, Japan) using an enzymatic colorimetric method.

### 2.1. Serum sNOX2-dp Detection

NOX-2-derived peptide (sNOX2-dp) release is a marker of NADPH oxidase activity and was evaluated in serum by an ELISA method as previously described [21]. Briefly, the assay is based on coating of reference standards and samples into a 96-well plate for at least 12 h at 4 °C. After the incubation, an anti-sNOX2dp-horseradish peroxidase (HRP) monoclonal antibody against the amino acidic sequence (224–268) of the extracellular membrane portion of NOX2 is added. The enzyme activity is detected after the addition of the substrate 3,3′,5,5′-tetramethylbenzidine (TMB) and measured spectrophotometrically at 450 nm. Values related to sNOX2-dp concentration were expressed as pg/mL and intra- and inter-assay coefficients of variation were 5.2% and 6%, respectively.

### 2.2. Hydrogen Peroxide (H_2_O_2_) Production

Hydrogen peroxide (H_2_O_2_) production was evaluated using a colorimetric assay according to manufacturer instructions (Abcam, Cambridge, UK). The reaction product was measured spectrophotometrically at 450 nm and expressed as μM. The intra- and inter-assay CV were both <10%.

### 2.3. Serum 8-iso-PGF2α-III

8-iso-PGF2α-III concentration was detected in serum by an ELISA commercial kit (FineTests, Hubei, China). The values were expressed as pg/mL and intra- and inter-assay coefficients of variation were 5.8% and 5.0%, respectively. 

### 2.4. Soluble P-Selectin (sP-Selectin) Assay

sP-Selectin values were measured by an ELISA commercial kit (DRG International, NJ, USA). The values were expressed as ng/mL and the intra- and inter-assay coefficients of variation were 4.3% and 6.1%, respectively.

### 2.5. Serum Nitric Oxide (NO) Bioavailability

NO bioavailability was evaluated in serum by a colourimetric kit (Abcam, Cambridge, UK) that analyze the metabolic end products, i.e., nitrite and nitrate (NOx) (arbor assays). The values of NO were expressed as uM and the intra- and inter-assay coefficients of variation were 2.9 and 1.7%, respectively.

### 2.6. Serum Thromboxane B2 (TXB2)

Serum TXB2 was measured by an EIA commercial kit (Tema Ricerca, Italy) and as pg/mL. Intra- and inter-assay coefficients of variation for TXB2 EIA Kit were 4.0% and 3.6%, respectively. 

### 2.7. Serum LPS Assay 

LPS was measured by performing specific sandwich enzyme-linked immunosorbent assay (ELISA) (Hycult Biotechnology, Uden, The Netherlands). The kit had a concentration range of 0.04 to 10.0 EU/mL. 2.6.1. A Pierce LAL Chromogenic Endotoxin Quantitation Kit was used to detect Gram-negative bacterial endotoxins (Thermo Fisher Scientific Waltham, MA, USA). Bacterial endotoxin catalyzed the activation of a proenzyme in the modified Limulus Amebocyte Lysate (LAL), which catalyzed the splitting of p-Nitroaniline (pNA) from the colorless substrate, Ac-Ile-Glu-Ala-ArgpNA; the activation rate was proportional to the endotoxin concentration of the sample. The released pNA was photometrically measured at 405–410 nm after stopping the reaction. Absorbance and endotoxin concentration are linearly correlated in the 0.1–1.0 EU/mL range. 

### 2.8. Serum Zonulin

Serum zonulin was used as an intestinal permeability assay. Serum zonulin levels were measured using a commercial ELISA kit (Elabscience, Houston, TX, USA). Antibody specific for zonulin has been pre-coated onto a microplate and 100 μL of standards, and patient sera samples were added and incubated for 90 min at 37 °C. Then, a biotinylated detection antibody specific for zonulin and Avidin-Horseradish Peroxidase (HRP) conjugate was added to each microplate. Values were expressed as ng/mL; both intra-assay and inter-assay coefficients of variation were within 10%.

### 2.9. Statistical Analyses

Comparisons between groups were carried out by *t*-test. Data are presented as mean ± SD. Proportions and categorical variables were tested by the X2-test. Logistic multivariable analysis was performed to evaluate the variables associated with the offspring of patients with early myocardial infarction. Spearman test was used for the correlation analysis. Multiple linear regression analysis was performed using a forward selection method to determine the independent predictors of sNOX-2-dp. Statistical significance was defined as *p* < 0.05. Statistical analysis was executed with SPSS 18.0 software for Windows (SPSS Inc., Chicago, IL, USA).

### 2.10. Sample Size Calculation

The minimum sample size was computed with respect to a 2-tailed 1-sample Student *t* test, considering a difference for serum NOX-2 between the offspring of patients with early myocardial infarction and HS of 7 pg/mL; SDs homogeneous between groups SDs = 10 pg/mL; and type I error probability a = 0.05 and power 1-b = 0.90. This resulted in a minimum sample size of n = 43 per group. 

## 3. Results

Clinical characteristics of the offspring of patients with early myocardial infarction and HS are reported in Table 1.

No significant difference was found between the two groups for age, BMI, systolic and diastolic blood pressure and fasting blood glucose. Conversely, compared to HS, total cholesterol levels were higher in the offspring of patients with early myocardial infarction. Among the parents of the index group, early myocardial infarction occurred at 42.7 ± 5.7 years old in 41 males (42.4 ± 5.8 years old) and 5 females (mean age 45.0 ± 4.5 years old); fatal myocardial infarction occurred in 6 patients (4 males and 2 females, mean age: 38.7 ± 3.7 years). As previously reported [3], compared to HS, offspring of patients with early myocardial infarction had higher values of serum TXB2, isoprostanes and sNOX-2-dp (Table 1). To better analyze the role of oxidative stress and platelet activation, we evaluated the levels of serum H_2_O_2_ and p-selectin that, compared to controls, were higher in offspring of patients with early myocardial infarction than in controls. Conversely, NO bioavailability was lower in offspring of patients with early myocardial infarction (Table 1). 

We also evaluated serum LPS and zonulin, which were significantly higher in the offspring of patients with early myocardial infarction. 

Logistic regression analysis showed that the variables associated with offspring of patients with early myocardial infarction were serum LPS, TXB2 and isoprostanes (Table 2). 

**Figure 1:**
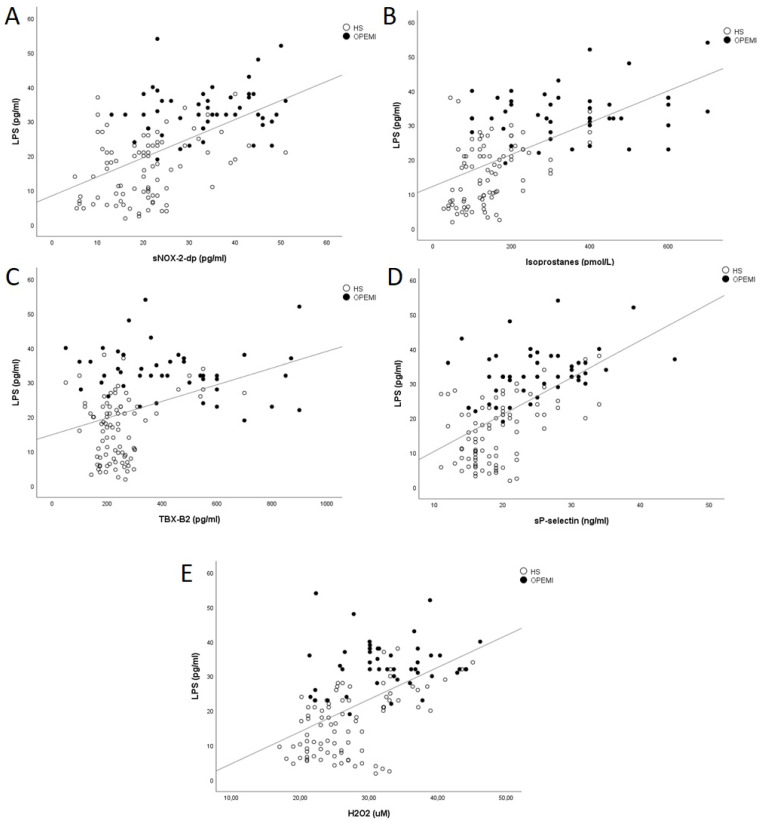
Simple linear regression analysis between serum LPS and NOX2 (**A**), isoprostanes (**B**), TXB2 (**C**), sP-Selectin (**D**) and H_2_O_2_ (**E**). OPEMI: offspring of patients with early acute myocardial infarction; HS: healthy subjects; LPS: lipopolysaccharide; NOX-2: NADPH oxidase-type 2; TXB2: Thromboxane B2, H_2_O_2_: Hydrogen Peroxide.

Bivariate analysis in the overall population showed that serum LPS was significantly associated with zonulin (Rs = 0.241; *p* = 0.005), sNOX-2-dp levels (Rs = 0.546; *p* < 0.001) (Figure 1A), serum isoprostanes (Rs = 0.607; *p* < 0.001) (Figure 1B), serum TXB2 (Rs = 0.368; *p* < 0.001) (Figure 1C), plasma sP-selectin (Rs = 0.575; *p* < 0.001) serum H_2_O_2_ (Rs = 0.561; *p* < 0.001) (Figure 1D,E), age (Rs = 0.228; *p* = 0.009) and total cholesterol (Rs = 0.230; *p* = 0.021). No significant correlation was found between LPS and NO bioavailability (Rs = −0.128; *p* = 0.142). Furthermore, sNOX-2-dp levels were associated with serum isoprostanes (Rs = 0.615; *p* < 0.001), serum H_2_O_2_ (Rs = 0.270; *p* = 0.002), serum TXB2 (Rs = 0.455; *p* < 0.001), plasma sP-selectin (Rs = 0.374; *p* < 0.001), serum zonulin levels (Rs = 0.225; *p* = 0.09) and total cholesterol (Rs = 0.203; *p* = 0.042). No significant correlation was found between total cholesterol and LPS in the group of offspring of patients with early myocardial infarction (Rs = 0.047; *p* = 0.756).

A multiple linear regression analysis, including the variables linearly associated with the dependent variable, was performed to define the independent predictors of serum LPS and sNOX-2-dp.

Serum sNOX-2-dp (SE: 0.76; standardized coefficient β: 0.176; *p* = 0.02), isoprostanes (SE: 0.006; standardized coefficient β: 0.308; *p* < 0.001), p-selectin (SE: 1.28; standardized coefficient β: 0.263; *p* < 0.001) and H_2_O_2_ (SE: 0.115; standardized coefficient β: 0.264; *p* < 0.001) were significantly associated with LPS (R2: 0.58).

Serum LPS (SE: 0.82; standardized coefficient β: 0.227; *p* = 0.007), isoprostanes (SE: 0.06; standardized coefficient β: 0.392; *p* < 0.001) and TXB2 levels (SE: 0.05; standardized coefficient β: 0.219; *p* = 0.003) were significantly associated with sNOX-2-dp (R2: 0.47).

## 4. Discussion

This study shows that offspring of patients with early myocardial infarction have increased low grade endotoxemia and that LPS is closely associated with NOX-2 activation. 

Previous studies showed that LPS was increased in the systemic circulation of adult patients with acute myocardial infarction [12,22] and coronary microvascular angina [16].

LPS is a glycolipid component of the external cell wall of Gram-negative bacteria and it has a pathogen-associated molecular pattern. LPS is recognized by innate immune system components such as Toll-Like Receptors (TLRs), a component of the family of highly conserved membrane pattern-recognition receptors [23].

TLRs are expressed ubiquitously; they recognize microbial components and bind them. This link leads to a cascade of events which include the activation of several immunological patterns and at the end the activation of the inflammatory response. LPS is considered a pro-atherogenic molecule for its mechanisms of transport, diffusion into the circulation and inflammatory damage. In physiological conditions, the intestinal barrier prevents the transport of bacterial components into systemic circulation. This occurs by several mechanisms, but in the end, three levels of defense can be identified: a chemical barrier, composed of antimicrobial peptides, secretory immunoglobulins and the mucus layer; an immunological barrier, formed by the gut-associated lymphoid tissue (GALT); and a physical barrier, formed by the epithelial tight junctions. The tight junction proteins regulate the paracellular passage of various substances, including pathogens [24]. In this study, we focused on serum zonulin as a marker of gut dysbiosis. Zonulin is a protein that increases gut permeability and is a modulator of the intestinal innate immunity: it is involved in the regulation of the tight junction activity, and in this way it regulates the movement of fluids, molecules and cells from the intestinal lumen to the bloodstream [12]. In the case of gut dysbiosis, the disruption of intestinal barrier function can foster the passage of LPS across the intestinal barriers causing low-grade systemic inflammation and oxidative stress, leading finally to vascular injury [12,25]. Furthermore, animal models showed that LPS induces ROS production through TLR4/NOX-2 activation [25,26]. LPS can activate NADPH oxidase in endothelial cells, platelets and macrophages increasing oxidative stress and inflammation (see graphical abstract). 

NO is one of the primary mediators of endothelium dependent vasodilatation and is generated via up-regulation of both endothelial nitric oxide synthase (eNOS) and inducible nitric oxide synthase (iNOS) [27]. In the vessel walls, NO may be scavenged by an elevated level of ROS produced primarily by NADPH oxidase [28]. 

Moreover, tetrahydrobiopterin (BH4), an important cofactor for endothelial nitric oxide synthase activity, plays a pivotal role in maintaining normal endothelial function [29]. In fact, under conditions of oxidative stress, BH4 can be oxidized to dihydrobiopterin (BH2), and eNOS becomes uncoupled, producing reactive oxygen species rather than NO. This results in a decrease of NO bioavailability and causes endothelial dysfunction [30,31,32]. This delicate balance has a critical role in the capacity of blood vessels to maintain normal homeostasis and is a common characteristic in vascular disease [33].

Accordingly, we show that serum nitrite/nitrate, a marker of NO generation, is decreased in offspring of patients with early myocardial infarction and is inversely associated with endotoxemia. 

As previously reported, NOX-2 is overactivated in offspring of patients with early myocardial infarction and is associated with increased serum levels of isoprostanes and thromboxane B2 [3]. LPS increases platelet activation when combined with collagen or ADP. This process leads to increased platelet H_2_O_2_ production, NOX-2 activation, generation of thromboxane and 8-iso-PGF2α-III [6]. The high levels of TXB2, sP-selectin and their association with LPS could support the association between platelet activation and low grade endotoxemia. The high levels of TXB2 and its close association with LPS could confirm the association between platelet activation and low grade endotoxemia. Thus, a physio-pathological hypothesis could be that an increase in systemic LPS in offspring of patients with early myocardial infarction activates NOX-2 and increases oxidative stress and platelet activation (Graphical abstract) [34]. The resulting systemic inflammation and the increased susceptibility of platelets to aggregation could contribute to the higher cardiovascular risk of these patients. Future studies should clarify whether the decrease in systemic LPS will be accompanied by a reduction in oxidative stress and platelet activation. The cause of increased systemic LPS levels was not investigated. Hypertension, elevated cholesterol, insulin resistance and obesity could contribute to low grade endotoxemia, increasing the oxidative stress and the risk of cardiovascular disease, as reported in different studies on adults [14,35,36,37,38,39] and subjects over 18 years old [40,41]. Few studies reported high levels of markers of endotoxemia and gut permeability in children with nonalcoholic fatty liver disease and/or obesity [42,43]. Another study by Manco et al. in overweight and obese children found that LPS was associated with arterial stiffness and it was proposed to have a direct role in producing vascular inflammation [44]. We cannot exclude that some risk factors may have affected the low grade endotoxemia in offspring of patients with early myocardial infarction. LPS is integrated into newly synthesized chylomicrons and is transported from gut into the circulation by pro-atherogenic lipoproteins, such as very-low-density lipoproteins (VLDL) and low-density lipoproteins (LDL). Some conditions, such as a high-fat meal or hypercholesterolemia, may promote increased endotoxemia [45]. Previous studies showed that high-density lipoproteins (HDL) can mitigate LPS-induced damage, so pathologic conditions, such as low HDL and high total cholesterol levels, could favor LPS-mediated inflammation [45]. Thus, increased levels of cholesterol in our patients might have favored this process [46].

Our study supports and extends previous data published by Lanza et al., who demonstrated an increased platelet reactivity in children of patients with early myocardial infarction [47]. They showed that offspring of patients with early myocardial infarction have a higher platelet reactivity. Furthermore, the authors studied the endothelial function. They discovered a reduced FMD in offspring of patients with early myocardial infarction but failed to demonstrate a relationship between endothelial dysfunction and increased platelet reactivity, suggesting that the mechanisms responsible for the two abnormal findings can largely be independent. 

Our study suggests a partial but potential explanation of higher oxidative stress and platelet activation observed in offspring of patient with early myocardial infarction, involving the LPS translocation due to an increased gut permeability. The consequent low grade endotoxemia could generate oxidative stress and platelet activation increasing the cardiovascular risk of these subjects. However, further follow-up studies are needed to investigate the association between LPS, oxidative stress and cardiovascular risk in children of stroke patients at an early age.

## 5. Limitations

This study has some limitations. First, it does not fully clarify the mechanism accounting for platelet activation. Second, different NADPH isoforms, such as NOX1 and NOX4, which could contribute to increased oxidative stress, were not evaluated. Third, we did not analyze the mechanism of LPS translocation from the gut microbiota to the systemic circulation. In the offspring of patients with early myocardial infarction, we observed increased serum zonulin and LPS levels with a significant correlation between them, which reflect LPS translocation. However, zonulin is an indirect marker of gut permeability and we cannot exclude the possibility that serum LPS could also derive from other sources, thereby further studies are needed to support this hypothesis. Moreover, dietary changes can induce alteration of gut microbiota composition. In particular, the Mediterranean diet can modulate the gut microbiota, increasing its diversity, while Western countries’ diet is associated with increased gut permeability [48]. We did not analyze the type of diet; thus, we cannot exclude that a high–saturated fat diet increased low grade endotoxemia in the offspring of patients with early myocardial infarction. Future studies are needed to analyze the role of diet or the use of probiotics in these patients to evaluate a possible reduction of systemic LPS and oxidative stress. Fourth, we did not perform echocardiogram and FMD as a measurement of cardiac and early endothelial dysfunction.

## 6. Conclusions

In conclusion, this study shows that offspring of patients with early myocardial infarction have a low degree of endotoxemia. This could activate NADPH oxidase-2, generating oxidative stress and increased platelet activity (see graphical abstract). Future studies are necessary to understand the physio-pathological role of dysbiosis in these patients in enhancing the cardiovascular risk.

## Figures and Tables

**Table 1:** Clinical features of offspring of patients with early acute myocardial infarction and HS.

Variables	Offspring of Patients with Early Acute Myocardial Infarction (N = 46)	Healthy Subjects (N = 86)	*p* Value
Age (years)	13.76 ± 6.858	11.73 ± 5.779	0.092
Gender (M/F)	26/20 (56.5/43.5%)	58/28 (67.4/32.6%)	0.214
Serum TXB2 ^a^ (pg/mL)	429.35 ± 222.859	243.01 ± 102.713	<0.001
8-iso-PGF2α-III (pg/mL)	352.46 ± 163.576	144.84 ± 83.649	<0.001
sP-Selectin (ng/mL)	26.13 ± 6.45	18.50± 4.30	<0.001
sNOX-2-dp ^b^ (pg/mL)	33.83 ± 10.369	20.35 ± 9.166	<0.001
H_2_O_2_ µM	34.33 ± 6.06	26.09 ± 5.17	<0.001
NO bioavailability µM	28.72 ± 7.24	47.28± 16.54	<0.001
Serum LPS ^c^ (pg/mL)	33.33 ± 7.242	16.43 ± 9.094	<0.001
Serum zonulin (ng/mL)	2.1533 ± 0.805	1.566 ± 0.666	<0.001
Total Cholesterol (mg/dL)	190.67 ± 49.58	170.69 ± 49.58	0.017
Glycemia (mg/dL)	86.13 ± 6.27	88.96 ± 11.07	0.136
Systolic blood pressure (mmHg)	110.78 ± 12.74	110.02 ± 9.77	0.746
Diastolic blood pressure (mmHg)	63.41 ± 9.66	66.79 ± 13.44	0.144
BMI ^d^	21.61 ± 4.38	20.76 ± 4.30	0.285

^a^ TXB2, Thromboxane B2; ^b^ sNOX-2-dp soluble NADPH oxidase-type 2 derived peptide; ^c^ LPS, lipopolysaccharide; ^d^ BMI, body mass index.

**Table 2:** Binary logistic regression analysis to assess the variables associated with offspring of patients with early acute myocardial infarction.

	O.R.	95% C.I.		
		Inferior	Superior	*p*
TXB2	1.008	1.001	1.014	0.023
Isoprostanes	1.013	1.002	1.024	0.022
LPS	1.281	1.119	1.467	<0.001

Non-significant variables in the model: sNOX2-dp (*p* = 0.499), Zonulin (*p* = 0.185), H_2_O_2_ (*p* = 0.754), sP-Selectin (0.425), NO (*p* = 0.456), total cholesterol (*p* = 0.469).

## Data Availability

Data will be available upon reasonable request to the corresponding author.
